# The changing multiple sclerosis treatment landscape: impact of new drugs and treatment recommendations

**DOI:** 10.1007/s00228-018-2429-1

**Published:** 2018-02-10

**Authors:** Irene Eriksson, Joris Komen, Fredrik Piehl, Rickard E. Malmström, Björn Wettermark, Mia von Euler

**Affiliations:** 1 Department of Medicine Solna, Karolinska Institutet, Stockholm, Sweden; 2 Department of Healthcare Development, Stockholm County Council, Stockholm, Sweden; 3 Department of Pharmaceutical Sciences, Utrecht University, Utrecht, the Netherlands; 4 Department of Clinical Neuroscience, Karolinska Institutet, Stockholm, Sweden; 5 Clinical Pharmacology, Karolinska University Hospital, Stockholm, Sweden; 6 Department of Clinical Science and Education, Södersjukhuset, Karolinska Institutet, Stockholm, Sweden

**Keywords:** Rituximab, Dimethyl fumarate, Drug and Therapeutics Committee, Drug utilization, Multiple sclerosis, relapsing-remitting

## Abstract

**Purpose:**

The purpose of this study is to describe the utilization of disease-modifying treatments (DMTs) in relapsing-remitting multiple sclerosis (MS) and assess the impact of both the introduction of new drugs and treatment recommendations (local recommendation on rituximab use issued at the largest MS clinic in Stockholm and regional Drug and Therapeutics Committee (DTC) recommendation on how dimethyl fumarate should be used).

**Methods:**

Interrupted time series analyses using monthly data on all MS patients treated with DMTs in the Stockholm County, Sweden, from January 2011 to December 2017.

**Results:**

There were 4765 individuals diagnosed with MS residing in the Stockholm County from 2011 to 2017. Of these, 2934 (62%) were treated with an MS DMT. Since 2011, fingolimod, alemtuzumab, teriflunomide, dimethyl fumarate, peginterferon beta-1a, and daclizumab were introduced. Only fingolimod and dimethyl fumarate significantly impacted MS DMT utilization. In parallel, the use of rituximab off-label increased steadily, reaching 58% of all DMT-treated MS patients by the end of the study period. The local recommendation on rituximab was associated with an increase in rituximab use. The regional DTC recommendation on dimethyl fumarate was associated with a decrease in dimethyl fumarate use.

**Conclusions:**

Three MS DMTs—fingolimod, dimethyl fumarate, and rituximab off-label—impacted MS DMT utilization in the Stockholm County. The associations between the treatment recommendations and the subsequent changes in MS DMT utilization indicate that such interventions can influence the uptake and utilization of new drugs used in the specialized care setting.

**Electronic supplementary material:**

The online version of this article (10.1007/s00228-018-2429-1) contains supplementary material, which is available to authorized users.

## Introduction

In the early 1990s, interferon beta emerged as the first disease-modifying treatment (DMT) for relapsing-remitting multiple sclerosis (MS). Since then, several interferon beta products were introduced that, along with glatiramer acetate, became the mainstay of MS treatment [1]. In 2006, natalizumab was launched for highly active relapsing-remitting MS and, since 2011, an additional eight DMTs were approved in Europe. Of these, fingolimod, teriflunomide, and dimethyl fumarate—the first oral DMTs—represented a long-awaited breakthrough, because for almost 20 years, only DMTs requiring parenteral administration had been available [2]. In addition, rituximab, approved for certain types of cancers and rheumatoid arthritis, has been increasingly used off-label to treat MS [3].

In Sweden, all MS DMTs (including rituximab off-label) are available to patients as treatment options (Online Resource 1). The choice of treatment is largely at the discretion of the treating neurologist. There are also non-binding recommendations—typically issued by the local clinics, professional associations, or by regional Drug and Therapeutics Committees (DTCs)—that aim to facilitate the rational use of drugs. In the Stockholm County, the largest region of Sweden, there have been two recent recommendations that focused on individual DMTs. In November 2012, the largest MS clinic in Stockholm issued a local recommendation that included rituximab as a treatment alternative for highly active MS. In October 2015, the regional DTC of the Stockholm County issued a recommendation on how dimethyl fumarate should be used in the region. While more expensive than interferon betas, dimethyl fumarate was perceived to be more effective and it was therefore recommended to channel its use to younger patients who in general have higher inflammatory disease activity [4].

In Stockholm, and elsewhere, there has been no thorough description of how the MS treatment landscape has changed following the recent introduction of new MS DMTs. More generally, it is of interest to explore how treatment recommendations impact the uptake and utilization of specialist drugs, particularly given the limited research in this area [5, 6]. The present study, therefore, described MS DMT utilization in the Stockholm County and assessed the impact of both the introduction of new drugs and the local and regional treatment recommendations.

## Methods

This is a population-based study of all Stockholm County residents diagnosed with MS and treated with DMTs from 1 January, 2011, to 31 December, 2017.

### Data sources

All data were derived from a regional data warehouse (VAL) in the Stockholm County that collects health-related data for all Stockholm County residents (2.3 million; approximately 23% of the population of Sweden) [7].

We used hospital discharge (inpatient) and outpatient specialist visit data to obtain information on diagnoses [International Classification of Diseases (ICD)-10] and procedures [Swedish Classification of Health Interventions and the Nordic Medico-Statistical Committee (NOMESCO) codes] from 1 January, 2010, to 31 December, 2017.

Outpatient drug utilization records were derived from an outpatient pharmacy dispensing database. DMTs administered in hospitals were identified using procedure codes and drug codes [Anatomical Therapeutic Chemical (ATC) classification]. These data have previously been validated using electronic health records [8]. Data on outpatient drug utilization were derived from 1 July, 2010, to 31 December, 2017. Data on inpatient drug utilization were derived from 1 January, 2010, to 31 December, 2017.

### Participants

We selected all patients with at least one MS diagnosis (ICD-10 code G35), either in inpatient or in outpatient specialist care, and at least one dispensation or administration of DMTs from 1 January, 2011, to 31 December, 2017. The DMTs available in the Stockholm County during the study period were interferon beta-1a, peginterferon beta-1a, interferon beta-1b, glatiramer acetate, natalizumab, fingolimod, alemtuzumab, dimethyl fumarate, teriflunomide, daclizumab, as well as rituximab though not formally approved as an MS DMT (Online Resource 1).

### Interventions

In our analyses, we aimed to study the impact of the following interventions: (1) the introductions of fingolimod (August 2011), alemtuzumab (September 2013), dimethyl fumarate (May 2014), teriflunomide (June 2014), peginterferon beta-1a (May 2015), and daclizumab (February 2017); (2) the local recommendation on rituximab issued at the largest MS clinic in Stockholm (November 2012); and (3) the regional DTC recommendation on dimethyl fumarate (October 2015). Online Resource 1 provides information on these and other key events since 2011 that may have influenced the utilization of DMTs in the Stockholm County. We used the dates of inclusion into the reimbursement scheme for fingolimod, dimethyl fumarate, teriflunomide, peginterferon beta-1a, and daclizumab and the marketing authorization date for alemtuzumab as proxies for the dates of introduction of these DMTs to the market. The impact of the local recommendation on rituximab was assessed within the MS clinic issuing this recommendation.

### Outcomes

The study outcome was the count of monthly prevalent DMT users throughout the study period. Individuals with at least a 1-day supply within a given month were considered prevalent users during that month. Exposure to DMTs dispensed in outpatient pharmacies was assessed using the dispensation date together with the number of dispensed packages and the DMT-specific administration regimen. For DMTs administered in hospitals, exposure duration was derived by adding the duration of potential clinical benefit to the date of DMT administration.

### Statistical analyses

**Fig. 1:**
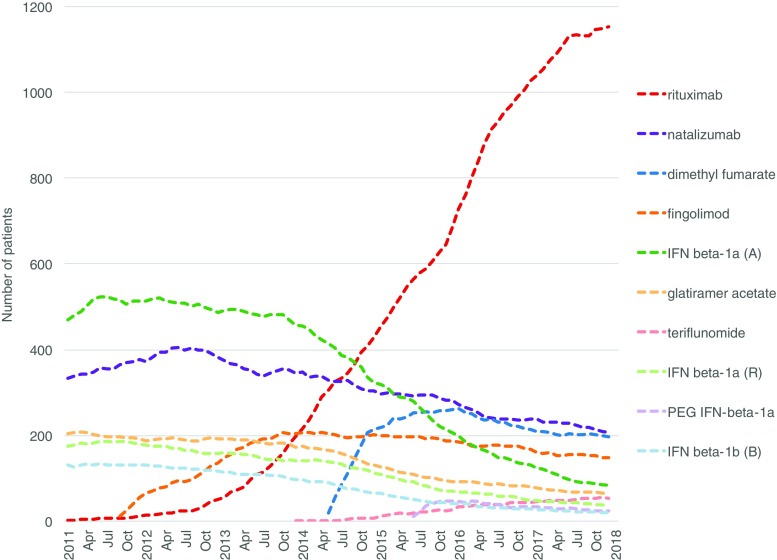
Number of prevalent MS DMT users from 2011 to 2017. A Avonex, B Betaferon, DMT disease-modifying treatment, IFN interferon, MS multiple sclerosis, PEG peginterferon, R Rebif. Legend is sorted by number of users in the month of December 2017. DMTs used by fewer than five patients are not shown

We carried out an interrupted time series analysis (ITS) [9]. First, we plotted the number of prevalent users each month for all DMTs (Fig. 1) in order to determine whether the different interventions could have potentially influenced utilization patterns. We only included interventions in the ITS analyses if they affected a DMT that was used by more than 5% of all users in any given month during the study period. We fitted a linear regression model over the time series for visual inspection of the time trends. With the Durbin-Watson statistic, we tested the data for first-order autocorrelation and corrected for this with an autoregressive term in the model if this was present [10, 11]. We used a segmented regression model with a step function to perform the ITS analysis [9, 12]. In this model, we included an indicator variable with the value of zero and one in the months before and after the intervention, respectively, to test for a step change in the number of users following the intervention.

With the time series model, baseline trends pre-intervention were analyzed and forecasted to estimate how the trends would continue if the intervention had not occurred [9]. We analyzed each intervention for two different outcomes, the step change (direct effect) and a change in slope (trend), both compared to the predicted values. When using an autoregressive model to correct for first-order autocorrelation, no linear forecasts were made, so the change in slope could only be assessed as significant or non-significant [10]. The magnitude and direction of the change in slope could be assessed with the linear regression models in the figures.

We chose pre- and post-intervention timeframes so that none of the other interventions overlapped with these time periods. When the step change clearly lasted longer than 1 month, we shaped the model to this.

## Results

During the study period (2011 to 2017), the Stockholm County had 4765 residents with at least one inpatient or outpatient diagnosis code for MS. Of these patients, 2934 (62%) received at least one dispensation or administration of an MS DMT. These patients comprised our study population (Online Resource 2).

Among the patients treated with MS DMTs in the beginning of 2011, half were treated with interferon beta-1a (Avonex and Rebif), 25% with natalizumab, 15% with glatiramer acetate, and 10% with interferon beta-1b (Betaferon). Fingolimod, introduced in September 2011, had a steady adoption and after the first 12 months on the market its share reached 6% of all DMT-treated patients. The uptake of dimethyl fumarate, first used in May 2014, was even faster (13% of the patients at 12 months). In parallel with the introduction of these new oral MS DMTs, the off-label use of rituximab was growing substantially. Already in October 2014, rituximab became the most commonly used DMT with a share of 22%. At the end of the study period, 58% of the DMT-treated patients were on rituximab, followed by natalizumab (10%), dimethyl fumarate (10%), and fingolimod (7%).

As can be seen in Fig. 1 and Online Resource 3, the other new MS DMTs—alemtuzumab, teriflunomide, peginterferon beta-1a, and daclizumab—were used in few patients. Neither accounted for more than 5% of the total MS DMT users in any given month. Therefore, only the introductions of fingolimod and dimethyl fumarate were included as interventions in the ITS analyses. Online Resource 4 shows the pre- and post-intervention timeframes of the included interventions, including the two treatment recommendations on rituximab and dimethyl fumarate.

**Fig. 2:**
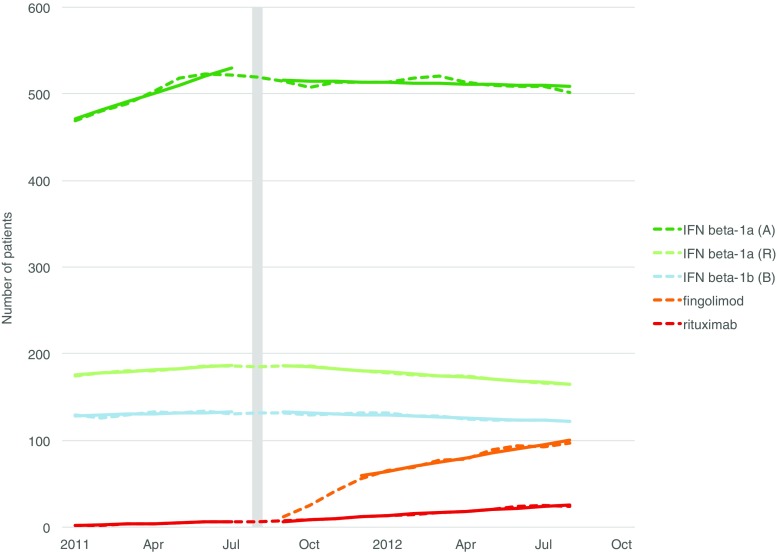
Association between the introduction of fingolimod and the number of DMT users. A Avonex, B Betaferon, DMT disease-modifying treatment, IFN interferon, R Rebif. Dashed lines are counts of prevalent users; solid lines are fitted models. The month of the intervention is indicated by the vertical bar. Only significant associations are shown

**Fig. 3:**
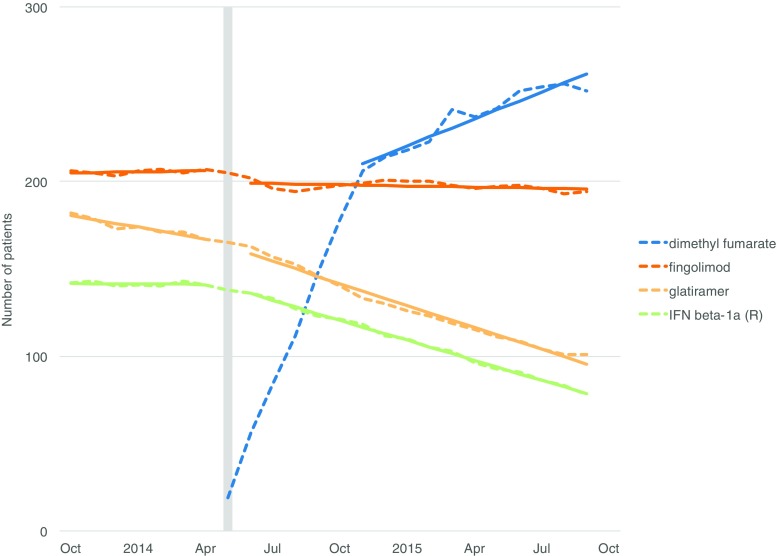
Association between the introduction of dimethyl fumarate and the number of DMT users. DMT disease-modifying treatment, IFN interferon, R Rebif. Dashed lines are counts of prevalent users; solid lines are fitted models. The month of the intervention is indicated by the vertical bar. Only significant associations are shown

**Fig. 4:**
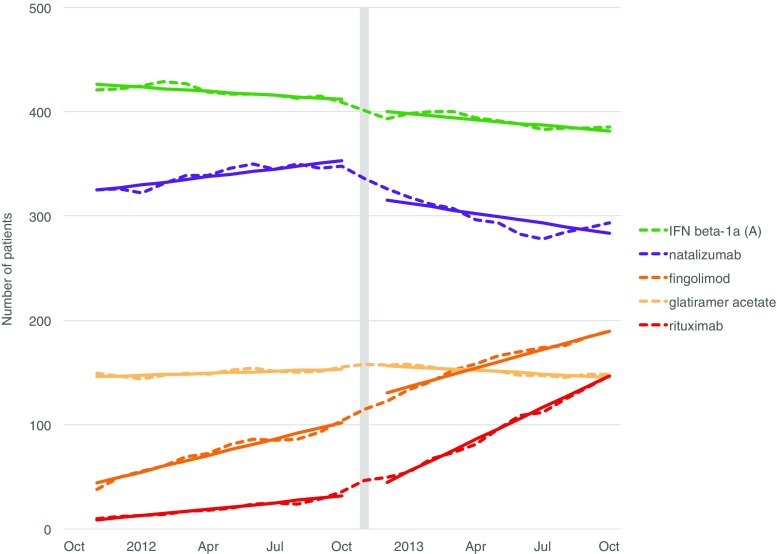
Association between the local recommendation on rituximab and the number of DMT users. A Avonex, DMT disease-modifying treatment. Dashed lines are counts of prevalent users (within the MS clinic issuing this recommendation); solid lines are fitted models. The month of the intervention is indicated by the vertical bar. Only significant associations are shown

**Fig. 5:**
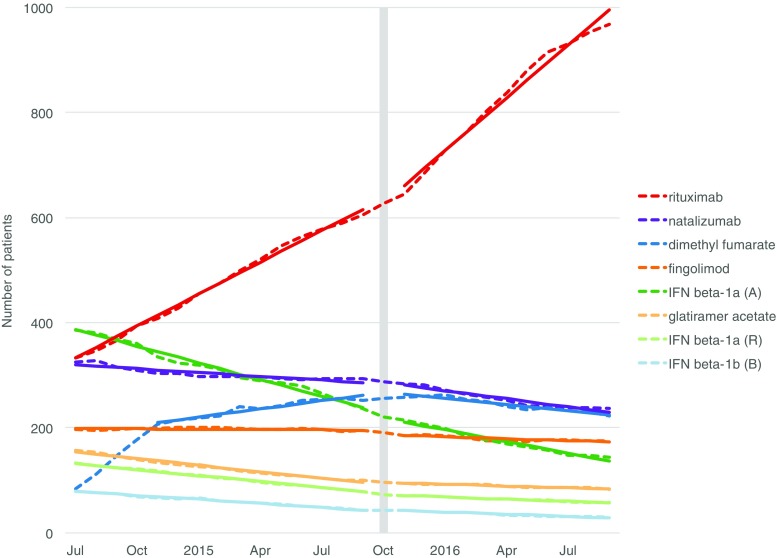
Association between the regional DTC recommendation on dimethyl fumarate and the number of DMT users. A Avonex, B Betaferon, DMT disease-modifying treatment, DTC drug and therapeutics committee, IFN interferon, R Rebif. Dashed lines are counts of prevalent users; solid lines are fitted models. The month of the intervention is indicated by the vertical bar. Only significant associations are shown

The data and the modeled associations between the interventions and the use of DMTs are illustrated in Figs. 2, 3, 4, and 5. Detailed results of the ITS analyses are provided in Online Resource 5.

The introduction of fingolimod was associated with changes in the trends of interferon beta-1a (Avonex and Rebif), interferon beta-1b (Betaferon), and rituximab (Fig. 2). The introduction of dimethyl fumarate was associated with changes in the trends of interferon beta-1a (Rebif) and glatiramer acetate (Fig. 3).

The local recommendation on rituximab was associated with an increase in rituximab use at the MS clinic that issued the recommendation (Fig. 4). Moreover, it was also associated with a decrease in natalizumab use as well as with a change in the trend of glatiramer acetate.

The regional DTC recommendation on dimethyl fumarate was associated with a significant change in the trends of all DMTs available at the time. While no direct effect was observed, use of dimethyl fumarate reached its peak soon after the recommendation and a clear downward trend followed.

## Discussion

This study demonstrates a considerable shift in MS DMT utilization. The number of available DMTs doubled from six (four interferon beta products, glatiramer acetate, and natalizumab) in early 2011 to 12 in 2017. In addition, the number of MS patients treated with rituximab grew steadily during the study period. In 2014, rituximab surpassed the other drugs to become the most used MS DMT in Stockholm.

Of the approved MS DMTs introduced since 2011, only fingolimod and dimethyl fumarate were being prescribed to a substantial number of MS patients, which may be explained by several factors. Anticipation and expectations for oral DMTs were high among patients and clinicians [13]. Oral administration promised an alternative for patients who experience adverse events associated with parenteral administration. It was also hoped that new oral DMTs would offer an advantage in effectiveness and tolerability compared to the conventional first-line treatment options (interferon betas and glatiramer acetate). Dimethyl fumarate was the first oral DMT approved for use as a first-line treatment option. While fingolimod—the first ever oral MS DMT—came to market years earlier, it had a more restricted label for patients with highly active MS. Teriflunomide—another oral DMT intended for first-line use—received an unfavorable initial reimbursement decision that allowed dimethyl fumarate to enter the MS DMT market with little competition. The initial reimbursement decisions for both teriflunomide and dimethyl fumarate were issued in May 2014 [14]. Dimethyl fumarate was included in the reimbursement scheme while teriflunomide faced an initial rejection. Shortly thereafter, however, teriflunomide was reimbursed for second-line use only. Incidentally, 2 years later, the reimbursement restriction was lifted and teriflunomide is currently the only oral DMT to be recommended by the regional DTC as a first-line treatment option along with the interferon betas [15].

The use of the other DMTs introduced during the study period was limited in our region. Extensive safety concerns, monitoring requirements, and a long duration of action resulted in alemtuzumab becoming a third-line treatment option. It is likely that the uptake would have been greater if autologous hematogenic stem cell transplantations (another third-line treatment option, not included in our analysis) and, in particular, rituximab had not been available. The limited uptake of peginterferon beta-1a can similarly be explained by the substantial use of rituximab, which has also been increasingly used as a first-line treatment option [16]. Finally, daclizumab saw almost no uptake with concerns about adverse events leading to the limited use. The largest MS clinic in the region participated in the clinical development program for daclizumab and had negative experiences with several cases of severe dermatological side effects.

Our study shows that both the local recommendation on rituximab and the regional DTC recommendation on dimethyl fumarate impacted utilization of the respective drugs as well as MS DMT utilization in general. The rapid adoption of rituximab as an MS DMT following the local recommendation issued at the largest MS clinic in Stockholm demonstrates the strong influence that clinicians, particularly in the specialized care setting, can have on the choice of treatments used. The decision to recommend rituximab was based on the growing knowledge of the comparative effectiveness of rituximab in relapsing-remitting MS [17] coupled with the well-established safety profile, a convenient administration regimen, and a relatively low cost. Perceived effectiveness and safety as well as convenience have previously been reported to be important factors influencing the choice of treatments in specialized care [5].

The decrease in the number of dimethyl fumarate users associated with the regional DTC recommendation is more difficult to interpret. Separate analyses showed that, as recommended, dimethyl fumarate prescribing shifted towards younger patients (52 and 72% of the new users were younger than 40 years before and after the recommendation, respectively). While our findings demonstrate a clear association between the DTC recommendation and the changes in the number of dimethyl fumarate users, there still may have been other factors leading to the decline in use. As the DTC recommendation was issued one and a half years after the introduction of dimethyl fumarate, clinicians may have already changed their prescribing patterns based on their experience with the drug by the time of the recommendation. We conducted ad hoc analyses to see if a similar drop in use appeared in the other large regions, but found the opposite—the use of dimethyl fumarate continued to increase (Online Resource 6). As we have no reasons to expect that the drug performed better in terms of effectiveness and safety in the other regions, we can assume that the DTC recommendation did have an impact in the Stockholm County. This finding is in line with results of a previous study showing an impact of DTC recommendations on the uptake and utilization of new drugs (non-vitamin K antagonist oral anticoagulants) [18]. In the current study, however, we assessed the uptake and utilization of drugs prescribed only by specialists who, compared to general practitioners, may respond differently to treatment recommendations [5, 19–21]. Moreover, specialists from the largest MS clinic in Stockholm (treating around 75% of all MS patients in the region) are affiliated with the regional DTC. This may contribute to the concordance between the regional DTC recommendation and the observed changes in MS DMT utilization.

This is, to the best of our knowledge, the first study to describe the utilization of new MS DMTs and to assess the influence of treatment recommendations on the use of these drugs. Our study has several strengths. First, we used an ITS design, which is the strongest quasi-experimental design in intervention research [9]. The ITS design has been shown to be appropriate for investigations of the effects of various interventions, and it has been used widely in drug utilization research [22]. Second, all data used in our study are population-based and prospectively recorded at the individual level. Third, our data include information on DMTs used in both the hospital and ambulatory settings for all MS patients treated in the region, thus providing a complete overview of drug utilization within the therapeutic area. Moreover, these data have been used previously in MS drug utilization studies and the hospital drug utilization records have been validated using electronic health records [7, 8].

The small number of patients in our study is a limitation. Because of this, we were not able to study changes in the incidence of prescribing and used the number of prevalent patients as the outcome. Using the count of incident users may have been more sensitive to change. Moreover, as there are few MS clinicians in our region and MS care is largely centralized to three university hospitals, a single prescriber may have impacted the overall MS DMT utilization. Also, this study is dependent on the accuracy and completeness of the information recorded in the databases we used. Finally, there may have been factors other than the studied interventions that influenced MS DMT utilization during the study period and that even could have coincided with the studied interventions.

To summarize, of all MS DMTs introduced since 2011, only fingolimod and dimethyl fumarate had considerable uptake and impacted MS DMT utilization in the Stockholm County. In parallel, rituximab, used off-label in MS patients, saw a steady increase in use over the entire study period and has been the most used MS DMT since 2014. The local recommendation on rituximab was associated with an increase in its use. The regional DTC recommendation on dimethyl fumarate also impacted MS DMT utilization and was associated with a decrease in the number of dimethyl fumarate users. These findings indicate that treatment recommendations—including those issued by DTCs—can influence the uptake and utilization of new drugs used in the specialized care setting.

## Electronic supplementary material

Online Resource 1 (PDF 47.6kb)

Online Resource 2 (PDF 356kb)

Online Resource 3 (PDF 107kb)

Online Resource 4 (PDF 19.2kb)

Online Resource 5 (PDF 39.3kb)

Online Resource 6 (PDF 24.5kb)
