# Supplementary material for: The changing multiple sclerosis treatment landscape: impact of new drugs and treatment recommendations
Source: Eur J Clin Pharmacol. 2018 Feb 10;74(5):663–70. doi: 10.1007/s00228-018-2429-1 (PMC5893684; doi:10.1007/s00228-018-2429-1)
Supplement: Supplementary file 1 — (PDF 47.6kb) [file 228_2018_2429_MOESM1_ESM.pdf]

MS DMTs available in the Stockholm County (2011–2017)

Active substance<br><br>Product name	Administration route<br><br>Frequency of administration	Marketing authorization*	Reimbursement#	Local and regional recommendations that mention treatment§
interferon beta-1b<br><br>Betaferon	Subcutaneous<br>3 times weekly	1995-11-30	Since before 2003	Wise List 2017¶
interferon beta-1a<br><br>Avonex	Intramuscular<br>Once weekly	1997-03-13	As above	Wise List 2017
interferon beta-1a<br><br>Rebif	Subcutaneous<br>3 times weekly	1998-05-04	As above	Wise List 2017
glatiramer acetate<br><br>Copaxone	Subcutaneous<br>Once daily or 3 times weekly	2001-09-21	2004-07-02	Wise List 2017
natalizumab<br><br>Tysabri	Intravenous<br>Once monthly	2006-06-27	2006-12-23	Wise List 2017
interferon beta-1b<br><br>Extavia	Subcutaneous<br>3 times weekly	2008-05-20	2008-10-01	—
fingolimod hydrochloride<br><br>Gilenya	Oral<br>Once daily	2011-03-17	2011-08-26	Wise List 2017
teriflunomide<br><br>Aubagio	Oral<br>Once daily	2013-08-26	2014-06-20 (restricted reimbursement)<br><br>2016-06-27 (general reimbursement)	Wise List 2017

**Active substance**<br><br>**Product name**	**Administration route**<br><br>**Frequency of administration**	**Marketing authorization***	**Reimbursement#**	**Local and regional recommendations that mention treatment§**
alemtuzumab<br><br>Lemtrada	Intravenous<br>Once yearly	2013-09-12	Hospital use	Wise List 2017
dimethyl fumarate<br><br>Tecfidera	Oral<br>Twice daily	2014-01-30	2014-05-09	2015-10-21— DTC treatment recommendation, Tecfidera should primarily be used in patients younger than 40 years with active RRMS without high inflammatory activity<br><br>Wise List 2017
peginterferon beta-1a<br><br>Plegridy	Subcutaneous<br>Twice monthly	2014-07-18	2015-05-22	Wise List 2017
daclizumab<br><br>Zinbryta	Subcutaneous<br>Once monthly	2016-07-01	2017-02-24 (restricted reimbursement)	—
rituximab<br><br>MabThera	Intravenous<br>Twice yearly	Not authorized for MS	Hospital use	2012-11-29 — Internal recommendations at the largest MS clinic in the region<br><br>2014-12-22 — Swedish MS Association recommendation<br><br>2016-12-15 — DTC treatment recommendations for RRMS§<br><br>Wise List 2017

DMT disease modifying treatment; DTC drug and therapeutics committee; MS multiple sclerosis; RRMS relapsing-remitting multiple sclerosis

*Medical Products Agency (www.lakemedelsverket.se)
#The maximum patient co-pay for prescription drugs (reimbursed drugs) is €220 during a 12-month period. Drugs administered in the outpatient setting are covered by the hospital/county council and the maximum patient co-pay for healthcare visits is €110 during a 12-month period. Before 2003-01-01, no exact reimbursement dates are available on the website of the Dental and Pharmaceutical Benefits Agency. For an overview of the Swedish system for pricing and reimbursement of pharmaceuticals, see *Pontén J, Rönnholm G, Skiöld P. PPRI Pharma Profile Sweden 2017. TLV Dental and Pharmaceutical Benefits Agency, 2017*.

§In December 2016, the National Board of Health and Welfare issued national MS guidelines (http://www.socialstyrelsen.se/publikationer2016/2016-12-1). Shortly thereafter, the Stockholm County DTC stated that their recommendations are in line with the national guidelines, with the addition of recommending rituximab as a first-line treatment option for MS patients with highly active RRMS.

¶The Wise List is the formulary for the Stockholm County Council, Sweden, which includes recommended essential medicines for common diseases in patients.
