# Supplementary material for: The changing multiple sclerosis treatment landscape: impact of new drugs and treatment recommendations
Source: Eur J Clin Pharmacol. 2018 Feb 10;74(5):663–70. doi: 10.1007/s00228-018-2429-1 (PMC5893684; doi:10.1007/s00228-018-2429-1)
Supplement: Supplementary file 2 — (PDF 356kb) [file 228_2018_2429_MOESM2_ESM.pdf]

Number of patients diagnosed with MS and treated with DMTs (2011–2017[*])

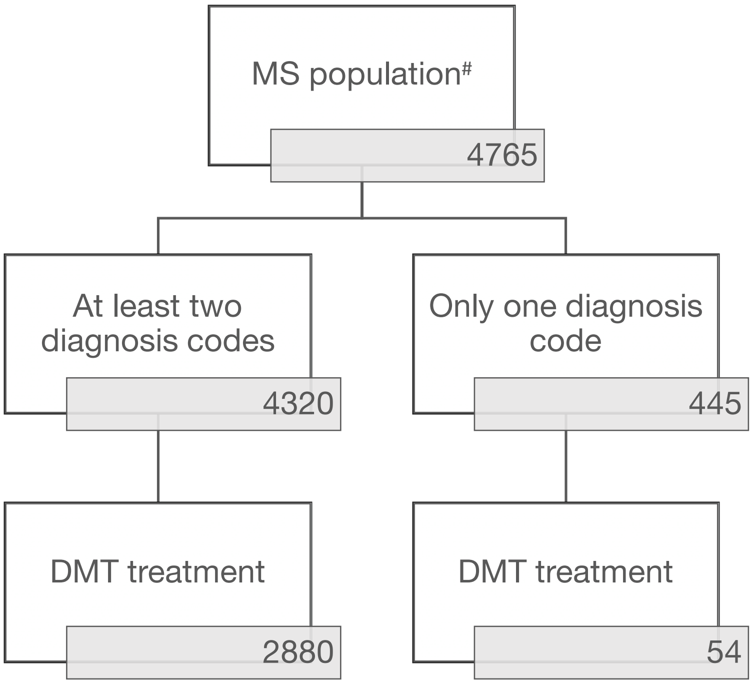

DMT	Number of patients[§]	Frequency (%)
Rituximab	1415	29
IFN beta-1a (Avonex)	853	18
Tysabri	762	16
Dimethyl fumarate	447	9
Fingolimod	357	7
Glatiramer acetate	353	7
IFN beta-1a (Rebif)	289	6
IFN beta-1b (Betaferon)	202	4
Teriflunomide	83	2
PEG beta-1a	78	2
IFN beta-1b (Extavia)	<5	–
Alemtuzumab	<5	–
Daclizumab	<5	–

DMT disease-modifying treatment; DTC drug and therapeutics committee; IFN interferon; MS multiple sclerosis; PEG peginterferon

*Study includes all residents of the Stockholm County between 1 January, 2011, and 31 December, 2017
#Patients with an MS diagnosis identified using inpatient or outpatient International Classification of Diseases (ICD)-10 code G35, recorded by a neurologist
§Patients with at least one at least one dispensation or an administration of an MS DMT and with an MS diagnosis identified using inpatient or outpatient ICD-10 code G35, recorded by a neurologist
