# Supplementary material for: The changing multiple sclerosis treatment landscape: impact of new drugs and treatment recommendations
Source: Eur J Clin Pharmacol. 2018 Feb 10;74(5):663–70. doi: 10.1007/s00228-018-2429-1 (PMC5893684; doi:10.1007/s00228-018-2429-1)
Supplement: Supplementary file 3 — (PDF 107kb) [file 228_2018_2429_MOESM3_ESM.pdf]

Number of prevalent MS DMT users from 2011 to 2017

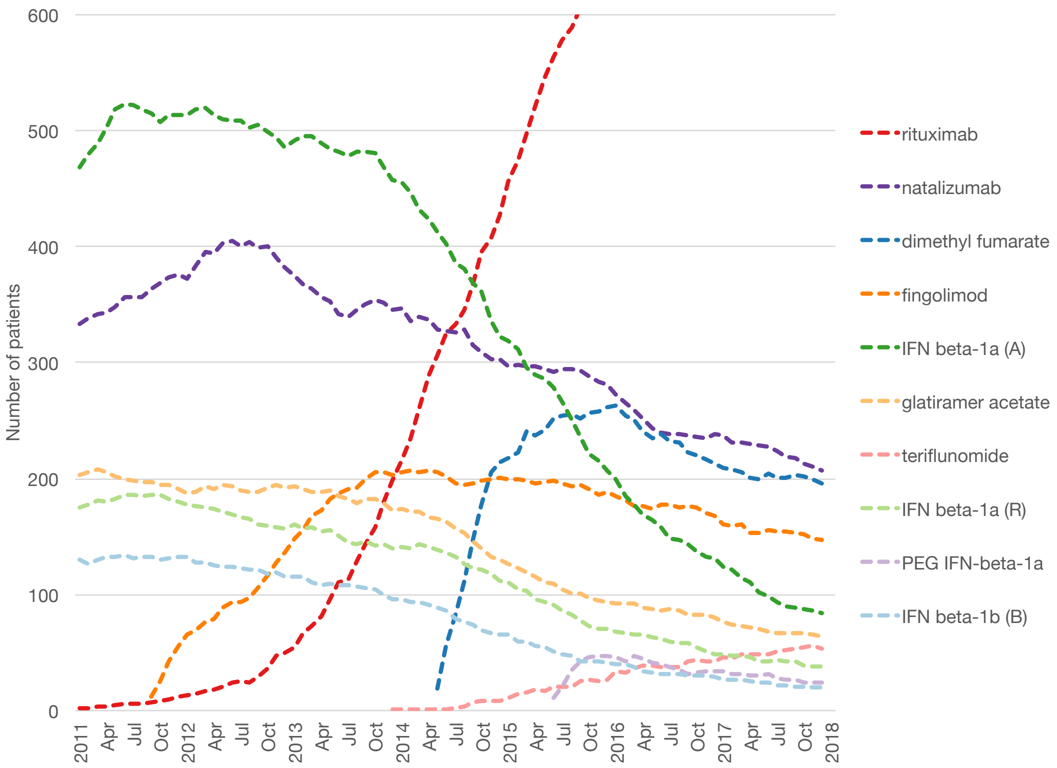

A Avonex; B Betaferon; DMT Disease-modifying treatment; IFN interferon; MS Multiple sclerosis; PEG Peginterferon; R Rebif. The y-axis is cut off at 600 for better visualization of the majority of the data (see Fig. 1 for a complete overview of drug utilization). The legend is sorted by number of users in the month of December 2017. DMTs used by fewer than 5 patients are not shown.
