# Supplementary figures and images for: The changing multiple sclerosis treatment landscape: impact of new drugs and treatment recommendations
Source: Eur J Clin Pharmacol. 2018 Feb 10;74(5):663–70. doi: 10.1007/s00228-018-2429-1 (PMC5893684; doi:10.1007/s00228-018-2429-1)

## Pre- and post-intervention timeframes for the included interventions

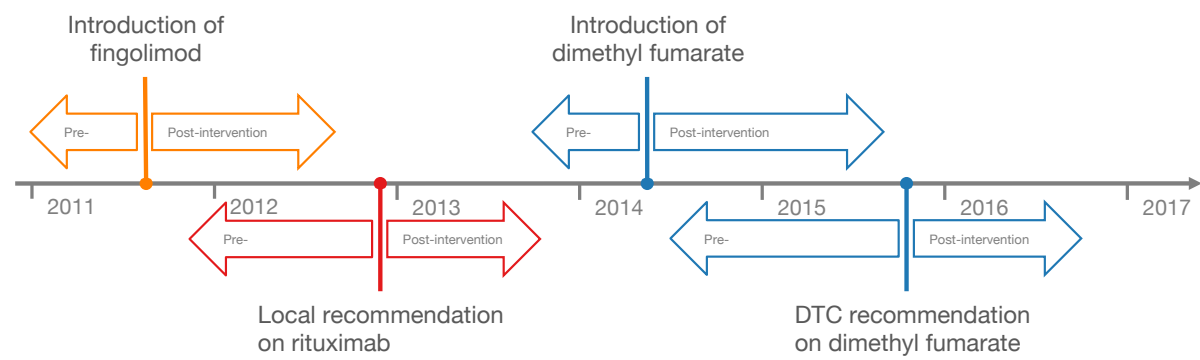

Supplement: Supplementary file 4 — (PDF 19.2kb) [file 228_2018_2429_MOESM4_ESM.pdf]
