# Supplementary material for: The changing multiple sclerosis treatment landscape: impact of new drugs and treatment recommendations
Source: Eur J Clin Pharmacol. 2018 Feb 10;74(5):663–70. doi: 10.1007/s00228-018-2429-1 (PMC5893684; doi:10.1007/s00228-018-2429-1)
Supplement: Supplementary file 5 — (PDF 39.3kb) [file 228_2018_2429_MOESM5_ESM.pdf]

**Associations between the studied interventions and the changes in the number of users of MS DMTs**

| Intervention | DMT* | Trend p-value | Level | Level p-value |
|---|---|---|---|---|
| Introduction of fingolimod 2011-08-26 | IFN beta-1b Betaferon | <0.001 | -1.9 | 0.342 |
| | IFN beta-1a Avonex | <0.001 | -32.2 | <0.001 |
| | IFN beta-1a Rebif | <0.001 | -5.2 | 0.001 |
| | Natalizumab | 0.568 | 2.1 | 0.653 |
| | Glatiramer acetate | 0.058 | -2.3 | 0.443 |
| | Fingolimod | <0.001 | 59.5 (3 months) | <0.001 |
| | Rituximab | <0.001 | 0.0 | 0.980 |
| Local recommendation (Karolinska University Hospital)# 2012-11-29 | IFN beta-1b Betaferon | 0.744 | 0.8 | 0.515 |
| | IFN beta-1a Avonex | 0.255 | -9.5 | 0.008 |
| | IFN beta-1a Rebif | 0.611 | 2.6 | 0.114 |
| | Glatiramer acetate | <0.001 | 1.8 | 0.328 |
| | Natalizumab | <0.001 | 42.9 | <0.001 |
| | Fingolimod | 0.219 | 17.6 | <0.001 |
| | Rituximab | <0.001 | 9.4 | 0.001 |
| Introduction of dimethyl fumarate 2014-05-09 | IFN beta-1b Betaferon | 0.079 | -4.2 | 0.030 |
| | IFN beta-1a Avonex | 0.188 | -9.0 | 0.150 |
| | IFN beta-1a Rebif | <0.001 | -5.1 | 0.001 |
| | Glatiramer acetate | 0.002 | -4.1 | 0.188 |
| | Natalizumab | 0.518 | -6.4 | 0.282 |
| | Fingolimod | 0.298 | -7.8 | 0.006 |
| | Dimethyl fumarate | <0.001 | 205.1 (5 months) | <0.001 |
| | Rituximab | 0.508 | -11.5 | 0.140 |

| Intervention | DMT* | Trend p-value | Level | Level p-value |
|---|---|---|---|---|
| DTC recommendation 2015-10-21 | IFN beta-1b Betaferon | <0.001 | 4.8 | 0.001 |
| | IFN beta-1a Avonex | <0.001 | -6.8 | 0.130 |
| | IFN beta-1a Rebif | <0.001 | 0.2 | 0.802 |
| | Glatiramer acetate | <0.001 | 7.7 | 0.001 |
| | Natalizumab | <0.001 | 0.1 | 0.977 |
| | Fingolimod | 0.002 | -9.9 | <0.001 |
| | Dimethyl fumarate | <0.001 | -7.9 | 0.091 |
| | Rituximab | <0.001 | 3.6 | 0.697 |

DMT disease-modifying treatment; DTC drug and therapeutics committee; IFN interferon; MS multiple sclerosis

*Only DMTs used by more than 5% of all users in any given month are shown
#Impact on utilization at Karolinska University Hospital only (accounting for about 75% of prevalent MS patients in the Stockholm County)
