# Supplementary material for: The changing multiple sclerosis treatment landscape: impact of new drugs and treatment recommendations
Source: Eur J Clin Pharmacol. 2018 Feb 10;74(5):663–70. doi: 10.1007/s00228-018-2429-1 (PMC5893684; doi:10.1007/s00228-018-2429-1)
Supplement: Supplementary file 6 — (PDF 24.5kb) [file 228_2018_2429_MOESM6_ESM.pdf]

Sales of dimethyl fumarate in the three largest regions of Sweden

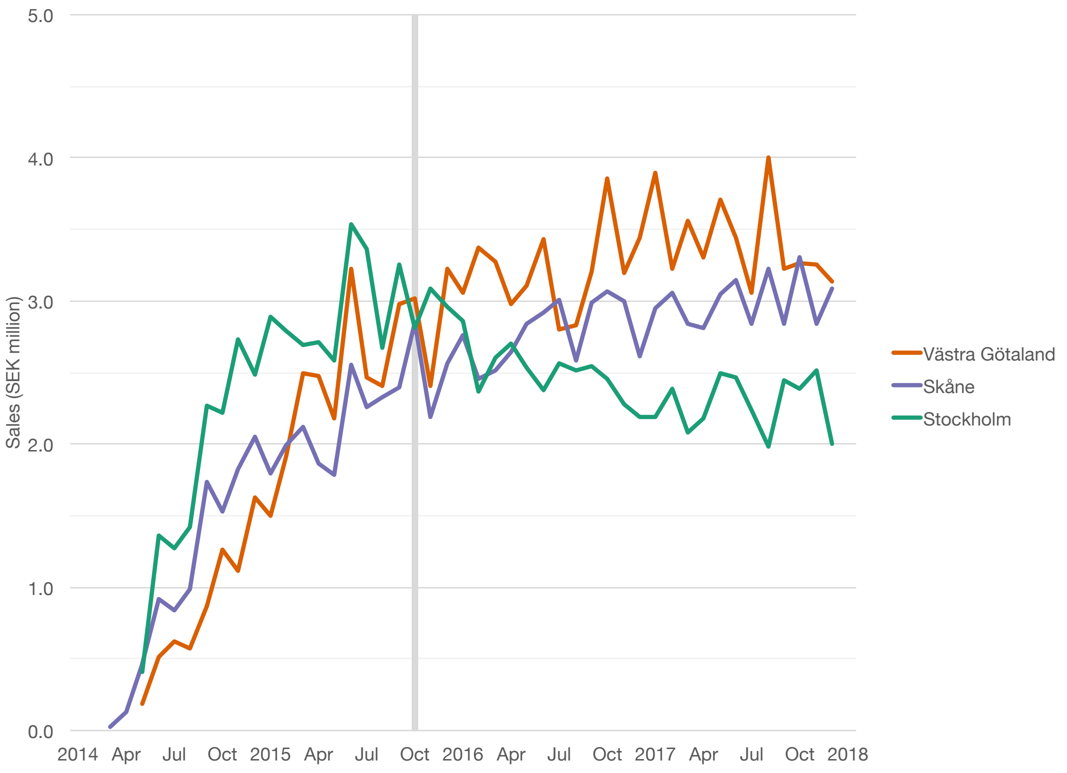

SEK Swedish krona. The month of regional Drug and Therapeutics Committee recommendation on how dimethyl fumarate should be used in the Stockholm County is indicated by the vertical bar.
